# Is advanced life support better than basic life support in prehospital care? A systematic review

**DOI:** 10.1186/1757-7241-18-62

**Published:** 2010-11-23

**Authors:** Olli-Pekka Ryynänen, Timo Iirola, Janne Reitala, Heikki Pälve, Antti Malmivaara

**Affiliations:** 1 University of Eastern Finland, Department of Public Health and Clinical Nutrition, P.O. Box 1627, 70211 Kuopio, Finland; 2 Kuopio University Hospital/Primary Health Care, P.O. Box 1777, 70211 Kuopio, Finland; 3 University of Turku and Turku University Hospital, Department of Anaesthesiology, Intensive Care, Emergency Care and Pain Medicine, P.O. Box 52, 20521 Turku, Finland; 4 Töölö Hospital, Helsinki University Central Hospital, Department of Anesthesiology and Intensive Care Medicine, P.O. Box 266, 00029 HUS, Finland; 5 Finnish Medical Association, P.O.Box 49, 00501 Helsinki, Finland; 6 Centre for Health and Social Economics, Insitute for Health and Welfare, Mannerheimintie 166, 00270 Helsinki, Finland

## Abstract

**Background -:**

Prehospital care is classified into ALS- (advanced life support) and BLS- (basic life support) levels according to the methods used. ALS-level prehospital care uses invasive methods, such as intravenous fluids, medications and intubation. However, the effectiveness of ALS care compared to BLS has been questionable.

**Aim -:**

The aim of this systematic review is to compare the effectiveness of ALS- and BLS-level prehospital care.

**Material and methods -:**

In a systematic review, articles where ALS-level prehospital care was compared to BLS-level or any other treatment were included. The outcome variables were mortality or patient's health-related quality of life or patient's capacity to perform daily activities.

**Results -:**

We identified 46 articles, mostly retrospective observational studies. The results on the effectiveness of ALS in unselected patient cohorts are contradictory. In cardiac arrest, early cardiopulmonary resuscitation and defibrillation are essential for survival, but prehospital ALS interventions have not improved survival. Prehospital thrombolytic treatment reduces mortality in patients having a myocardial infarction. The majority of research into trauma favours BLS in the case of penetrating trauma and also in cases of short distance to a hospital. In patients with severe head injuries, ALS provided by paramedics and intubation without anaesthesia can even be harmful. If the prehospital care is provided by an experienced physician and by a HEMS organisation (Helicopter Emergency Medical Service), ALS interventions may be beneficial for patients with multiple injuries and severe brain injuries. However, the results are contradictory.

**Conclusions -:**

ALS seems to improve survival in patients with myocardial infarction and BLS seems to be the proper level of care for patients with penetrating injuries. Some studies indicate a beneficial effect of ALS among patients with blunt head injuries or multiple injuries. There is also some evidence in favour of ALS among patients with epileptic seizures as well as those with a respiratory distress.

## Introduction

Prehospital care is an essential part of the treatment process in many acute diseases and trauma. Prehospital care is usually classified into ALS- (advanced life support) and BLS-(basic life support) treatment levels according to the methods used [1]. ALS refers to sophisticated prehospital care using invasive methods, such as intravenous fluids, medications and intubation. The vehicle used in ALS has either been a ground ambulance (GA) or a helicopter. Basic Life Support (BLS) is medical care which is used to assure patient's vital functions until the patient has been transported to appropriate medical care. ALS-level prehospital care has usually been implemented by physicians or paramedics, while BLS-level care is given by paramedics or emergency medical technicians. However, in most cases ALS units use the same techniques as BLS units.

While the concepts associated with ALS and BLS are diverse and differ between countries, both have developed towards greater sophistication. Some procedures that were previously classified as ALS-level prehospital care are now also available as part of BLS.

In spite of active research, the effectiveness of ALS care compared to BLS has been questioned [2]. Several research reports have been published, though no final conclusion has been drawn. Research projects have used different methods and target groups, and results have been controversial. The implementation of prehospital care is strongly dependent on local political, geographical, cultural and economic factors, making comparisons between systems difficult. The effectiveness of prehospital care also depends on the transportation method used and the emergency care given in the hospital. Thus, the problem of the effectiveness of ALS compared to BLS is only one link in the whole emergency care chain.

In emergency care, two alternative strategies have generally been presented [3]:

1. *scoop and run*: the patient is transported to a high level hospital as quickly as possible, with minimal prehospital treatments

2. *stay and play*: the patient is stabilized on site before transportation.

While debate on the merits of these two strategies is still ongoing, their division has been criticized for oversimplifying the problems of emergency care. Moreover, the two strategies do not correspond exactly to the division between ALS and BLS prehospital treatments. In the United States, the scoop and run strategy has been favoured, whereas in Europe several emergency systems use a stay and play -approach.

Researching and comparing studies in emergency care is difficult. Two main problems arise: Finding a suitable comparator across individual studies and also difficulties in comparing studies performed within different health care systems. ALS and BLS also entail different protocols in different countries.

Emergency care is affected by several elements:

• amount of population in an operational area

• geographical variables such as lakes, rivers, mountains

• quality and network of roads

• location and level of hospitals

• distribution of accident risk in the operational area

• amount, distribution, dispatching and quality of emergency units

• education of the personnel

• alarm systems

• communication technology, e.g. mobile phones, telemedicine

• development of the traffic: quality of vehicles and roads, traffic jams

The need for ALS procedures is quite rare and mostly ALS and BLS units provide the same levels of care. The factors influencing emergency care are not constant; they may change rapidly. The whole treatment chain can be totally different at night compared to the daytime.

The aim of this systematic review is to compare the effectiveness of ALS and BLS. The review covers all patient groups (e.g. trauma, cardiac disease, cardiac arrest, respiratory distress, convulsions) and all vehicles used for transportation of the team/patient (GA, helicopter, or both). The full report has been published in Finnish (available from: http://finohta.stakes.fi/EN/index.htm) [4].

## Methods

### Data Sources

The literature search was conducted from the following databases: PubMed, preMEDLINE OVID Medline, CRD databases, Cochrane database of systematic reviews, EBM reviews, CINAHL. To explore the grey literature, we made a search from the Internet by using Google Scholar search engine. The review period covered the years 1995-2008. All languages were included.

Combinations of the following search terms were used: advanced life support, basic life support, ALS (not amyotroph*), BLS, emergency medical services, emergency treatment, advanced cardiac life support, emergency, trauma, thrombolytic therapy, thromboly* fibrinoly*, prehospital, pre-hospital out-of-hospital (care or treatment or management or triage), paramedic, technician, ambulance*, helicopter, HEMS, mobile unit.

**Figure 1:**
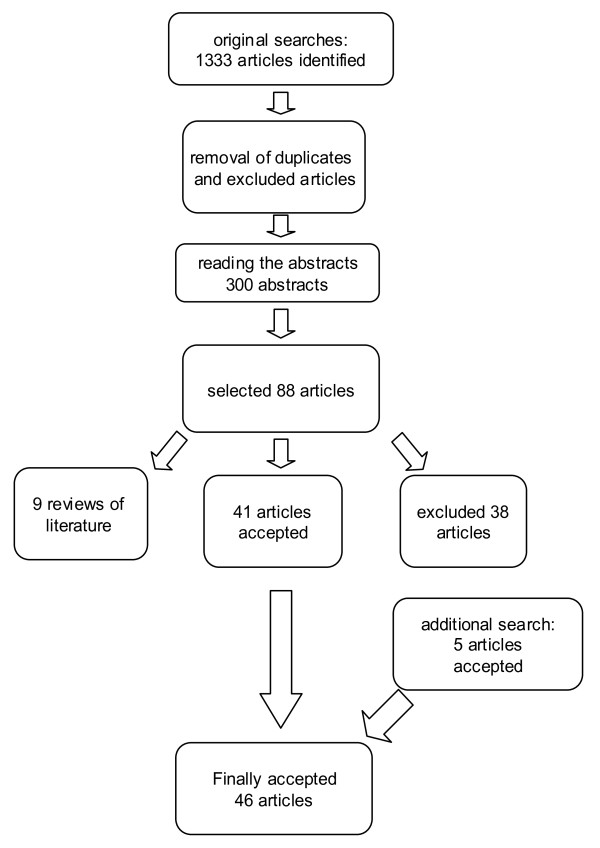
**Flow diagram of the search process**.

We performed a *related articles -*search from the PubMed for all articles we included after reading the abstracts. We also checked the reference lists from all relevant articles. The search process is presented in Figure 1.

We also prepared a general overview of the previous reviews on the effectiveness of prehospital care and helicopter emergency services. The search strategy was the same as that used in the literature search for the systematic review.

### Selection criteria

Articles were included if they fulfilled at least one of the following criteria:

1. ALS prehospital care was compared to the BLS, or

2. two different ALS systems were compared (e.g. physician-ALS compared to paramedic-ALS), or

3. ALS prehospital care was compared to any other treatment (e.g. ALS care compared to patient transport by laypersons).

4. A comparison between ALS and BLS was done virtually by an expert group.

The accepted outcome variables were:

1. Survival with a follow-up period until discharge from the hospital or later, or

2. Patient's health-related quality of life or capacity to perform daily activities at follow-up.

This systematic review is focused on patients' secondary survival. We accepted only the studies with a follow-up period until hospital discharge or later. We considered that studies using survival until arrival to the hospital are sensitive to the transport system and distance. We did not accept articles that only discussed treatment practices or treatment delays. Also we excluded articles using surrogate outcomes such as blood pressure or pain. Articles based on geographical epidemiology were also excluded.

Studies concerning thrombolytic therapy were included if the thrombolysis was given by a prehospital emergency care unit (e.g. in an ambulance). Thus, studies in which the thrombolysis was performed by a general practitioner were excluded.

#### Interventions

BLS was defined as a prehospital emergency service using non-invasive life-saving procedures including cardiopulmonary resuscitation, bleeding control, splinting broken bones, artificial ventilation, basic airway management and administration of oral or rectal medications. Use of a semi-automatic defibrillator was considered to be a part of BLS. Some BLS systems are allowed to use adrenaline in resuscitation. They were accepted as BLS if they were referred as a BLS in the article. BLS is usually provided by emergency medical technicians (EMT) or other similarly trained professionals.

ALS was defined as a prehospital emergency medical service using invasive life-saving procedures including all procedures of BLS but including advanced airway management, intravenous infusions and medications, synchronized cardioversion, cardiac monitoring, electrocardiogram interpretation and other procedures conventionally used at the hospital level. ALS is provided by physicians, paramedics or by other specially trained professionals.

### Data extraction

The following data were gathered from all the included articles: Bibliographical data (author, title, journal, year, volume, issue, pages), research aim, research methods (prospective, retrospective), years of gathering data, place of research (state or other), description of the research population, including patients' age and severity of disease (expressed as injury severity scale or other), professionals involved (physician, paramedic, EMT), transportation method, transportation time and distance, available and used treatments, treatment delays, baseline differences in the research population, mortality, health-related quality of life or capacity to perform daily activities, adjusted outcomes, transferability of research population and treatments across jurisdictions, amount of drop-outs and blinded measurement of outcome variables.

The articles were classified as follows:

1. Randomized controlled trials.

2. Studies where ALS care was compared to cases where ALS care was requested but not obtained.

3. Prospective studies using the TRISS-methodology or another comparable method to adjust the comparison between ALS and BLS or other control.

4. Epidemiological studies with a follow-up of all emergency patients and comparison between ALS and BLS.

5. Quasi-experimental studies with a comparison of ALS in one area with BLS in another area.

6. Before and after -studies using ALS and BLS data gathered in the same area at a different time.

7. Retrospective case-control studies using matched controls.

8. Studies based on expert panels.

The fulfilment of search criteria and the validity and methodological quality of all included studies were assessed by two independent researchers. In cases of controversy, a third researcher was consulted until a consensus was reached. Besides classification based on study design the following quality assessments of each individual study were made: unselected patients recruited, baseline differences, number of drop-outs described and blinded outcome assessment.

## Results

We found 1333 references from the databases. In additional searches, five articles were identified. Two researchers read the abstracts independently as well as identified the original articles. Altogether, 46 articles were included. Additionally, we identified eight previous meta-analyses or reviews [1,5-11]. The search process is presented in Figure 1. A summary of previous reviews is presented in Table 1 and original articles in Table 2[12-57].

**Table 1:** Summary of findings in the previous reviews on effectiveness of advanced vs. basic life support.

*Reference*	*Author(s) of review, year, country*	*description of a review*	*contents of the review*	*assessment of the review*	*conclusion*
5	Liberman et al. 2000, Canada	non-systematic review, traumas only	15 studies from years 1983-1997; classification according to quality: 1. medium quality 5 studies favouring ALS, 1 study favouring BLS. 2.high quality: 1 favouring ALS, 1 study favouring BLS 3. very high quality: 1 favouring ALS, 6 favouring BLS	In general the quality of studies was poor, many studies quite old, the follow-up periods starting even from 1930's.	7 studies favourintg ALS, 8 studies favouring BLS. Studies of higher quality favouring BLS.
6	Sethi et al. 2000 England	A systematic Cochrane-review		Only one study included	No difference between ALS and BLS
7	Nicholl et al. 2003, England	A systematic review on the effectiveness of helicopter services	36 original studies	HEMS better than ground transportation, mortality OR = 0,86, not statistically significant.	
8	Koskinen 2005, Finland	thesis for master's degree in health economics, contains a non-systematic review	36 original studies	In general the quality of studies was poor	cost-effectiveness of a helicopter service was assessed to be 5750 €/life year gained (confidence interval 2000 - 24500€)
9	Isenberg and Bissel, 2005, Canada	A non-systematic review, four parts: 1. trauma 2. cardiac arrest 3. cardiac infarct 4. distubances of consciousness	20 original articles, 2 meta-analyses from years 1984-2004	1. Trauma: 14 studies, 8 favouring ALS, 6 BLS. All new studies favouring BLS 2. cardiac arrest: early resuscitation and defibrillation associated with better survival, no special effect of ALS detected. 3. Cardiac infarct: 1 study, no difference between ALS and BLS. 4. Disturbances of consciousness, 1 study, no difference between ALS and BLS.	In general results favouring BLS. Review for paramedic-ALS only, physician-ALS excluded.
10,11	Thomas 2004, Thomas 2007	Qualitative review, renovation by a new version			No conclusion

**1**	Liberman 2007	Opinion-based article about trauma treatment, grounded by a non-systematic review			In general favouring BLS.

**Table 2:** Summary of findings in the articles presenting effectiveness of advanced vs. basic life support.

*Reference no*	*research, author, country, publication year*.	*type on the study* *illness or injury*. *research population*. *n(ALS), n (BLS)*. *severity of disease or injury*	*the implementer of the care*. *ALS, BLS*. *transport*. *ALS, BLS*. *treatments*.	*Outcome, mortality, other outcome variables, results*	*conclusion*
12	Shuster et al. 1995 Canada	prospective chart review. acute cardiac disease. ALS n = 1821 BLS n = 1245.	ALS-PARAMEDIC, GA. BLS-EMT, GA. an urban setting with short transportation times (less than 10 minutes)	mortality ALS 16,5% BLS 19,5% risk of death ALS OR = 1 BLS OR 1,12 (0,78-1,61)	no difference between the groups
13	Boissel 1995 France	multicentre study in 16 countries, PHT compared with thrombolysis in a hospital. ALS (immediate PTH) n = 2750. BLS (hospital throbolysis) n = 2719.	ALS-MD, GA. BLS-MD, GA. both groups treated by a physician.	30-day mortality ALS 9,7%, BLS 11,1% adjusted p = 0,08	trend to favour PHT (ALS).
14	Alldredge et al., California, U.S.A., 1995	retrospective chart review. children with status epilepticus, ALS n = 19 (treatment on site) BLS n = 26 (treatment in a hospital)	ALS-PARAMEDIC, GA BLS-EMT, GA prehospital diazepam therapy (given rectally or intravenously)	duration of status epilepticus ALS 32 min, BLS 60 min (p = 0,007) repeated cramps ALS 56%, BLS 85% (p = 0,045), mortality 0%	favours ALS.
15	Adams et al.1996 Illinois, U.S.A.	retrospective study. declined level of (epilepsy, hypoglycaemia, stroke). ALS n = 113, BLS n = 90	ALS-paramedic, GA BLS-EMT, GA	mortality ALS 6%, n = 7 BLS 2%, n = 2	no difference between the groups
16	Demetriades et al. 1996 California, U.S.A.	retrospective, all traumas ALS or BLS n = 4856 private transport n = 926	ALS-PARAMEDIC ori BLS-EMT compared with patients transported by a private vehicle	mortality: ALS or BLS 9,3% private transport 4,0% adjusted RR 1.60 (P = .002).	better survival and less permanently disabled in privately transported patients
17	Silfvast and Ekstrand 1996 Finland	before-after-design, prehospital cardiac arrest before (Period I, retrospective) and after (Period II, prospective) reorganisation of the EMS system. Phase I: ALS-PHYSICIAN experienced physicians, n = 444 Phase II: ALS-PHYSICIAN junior physicians, n = 395	two ALS-systems. physicians experienced (Phase I) and less experienced (Phase II), both operated with a GA	total mortality: Phase I 90.8% Phase II 91,6% (NS) survival of patients with ventricular fibrillation phase I: 41 (34%) phase II: 33 (25%) p = 0,05	no difference between groups in total mortality. among patients with ventricular fibrillation better results in phase I
18	Nguyen-Van-Tam et al. 1997 England	retrospective cohort cardiac arrest ALS n = 285 BLS n = 144 dual response n = 79	ALS-PARAMEDIC, BLS-EMT dual response: both ALs and BLS in the scene. GA in all groups.	mortality: ALS 91,9%, dual 98,7%, BLS 93,8%, p = 0,63) ALS adjusted survival RR 1,21 (0,50-2,91)	no difference between ALS, BLS and dual response groups
19	Rainer et al. 1997a England	prospective trauma patients ALS n = 247 BLS n = 843	ALS-PARAMEDIC, ALS-EMT ALS GA BLS GA	mortality: ALS 4%, BLS 3% (NS) TRISS: unexpected deaths: ALS n = 5, BLS n = 18 unexpected survivals: ALS n = 6, BLS n = 9, (NS)	no difference between ALS and BLS groups
20	Rainer et al. 1997b England	prospective cardiac arrest ALS n = 111 BLS n = 110	ALS-PARAMEDIC, BLS-EMT ALS GA BLS GA	mortality ALS 93%, BLS 94% p = 0,59. resuscitation by a bystander and early defibrillation associated with better survival	no difference between ALS and BLS groups
21	Suominen et al. 1998 Finland	retrospective pediatric trauma, ALS n = 49 BLS n = 72, total material n = 288	ALS-PHYSICIAN, BLS-EMT ALS helicopter and GA, BLS GA	ALS 22,4% BLS 31,9% (NS)	no difference between groups, a subgroup ISS 25-49 ALS better (p = 0,04)
22	Nicholl et al. 1998 Sheffield, England	retrospective trauma ALS n = 882 BLS n = 331	ALS-PARAMEDIC, BLS-EMT GA in both groups	6 months follow-up: mortality ALS 6,0%, BLS 4,6% OR 2,02 (1,05-3,89) ALS: higher mortality in penetrating trauma and large fractures	higher mortality in ALS
23	Eisen and Dubinsky 1998, Canada	retrospective all patient groups in prehospital care BLS n = 1000 ALS n = 397	ALS-PARAMEDIC (level 2 and level 3, level 1 = BLS), BLS-EMT GA in both	mortality: ALS 5,8%, BLS 4,6% (NS), LOS. no difference between groups	no difference between groups
24	Abbott et al. 1998 California U.S.A.	prospective case-control closed head injury ALS-PHYSICIAN n = 196 ALS-PARAMEDIC n = 1090 HEMS manned by nurse or nurse/paramedic/physician	ALS-HEMS ALS-PARAMEDIC ALS-PHYSICIAN helicopter ALS-PARAMEDIC GA	ALS-PHYSICIAN 20% ALS-PARAMEDIC 31% OR 1,75 1,21 - 2,53 subgroups: age, GCS had effect on mortality	ALS-HEMS better than ALS-PARAMEDIC
25	Owen et al. 1999 Texas, U.S.A.	retrospective TRISS trauma patients, comparison between helicopter and GA, ALS-PARAMEDIC (GA) n = 687 ALS-PARAMEDIC (helicopter) n = 105	ALS-PARAMEDIC (GA) ALS-PARAMEDIC, ALS-N (helicopter) ALS-PARAMEDIC (GA) ALS-PARAMEDIC, ALS-N (helicopter)	mortality: 14,3%, 6,0% TRISS: GA predicted 39 deaths, actually 41, helicopter: predicted 16 deaths, actually 15	no difference between groups
26	Mitchell et al. 2000 Scotlandi	before-after design cardiac arrest, period 1 n = 259 period 2 n = 294	ALS-PARAMEDIC, GA	period 1 94,2% period 2 93,5%	no difference between groups
27	Eckstein et al. 2000 California, U.S.A.	retrospective serious trauma ALS n = 93 BLS n = 403	ALS-PARAMEDIC, BLS-EMT, ALS GA, BLS GA	mortality ALS 93%, BLS 67% adjusted 5,3 (2,3 -14,2)	higher mortality in ALS
28	Pitetti et al. 2001 Pennsylvania, U.S.A.	retrospective pediatric cardiac arrest ALS-PARAMEDIC n = 150 BLS-EMT n = 39	ALS-PARAMEDIC BLS-EMT ALS GA, BLS GA	ALS 96,7% BLS 0% (NS)	no difference between ALS and BLS groups
29	Garner et al. 2001 Australia	retrospective comparison between two ALS-systems blunt trauma in head ALS-PARAMEDIC n = 250 ALS-PHYSICIAN n = 46	comparison of two levels of ALS ALS-PARAMEDIC GA ALS-PHYSICIAN helicopter (91%)	mortality: ALS-PHYSICIAN 20% ALS-PARAMEDIC 31% survival ALS-PARAMEDIC OR = 1, ALS-PHYSICIAN OR = 2,70 (1,48-4,95)	ALS-PHYSICIAN better than ALS-PARAMEDIC
30	Di Bartolomeo et al. 2001 Italy	ALS patients compared with cases when ALS was requested but not obtained Serious brain injury ALS-PHYSICIAN n = 92 BLS-H n = 92	ALS-PHYSICIAN helicopter BLS-H GA	mortality: ALS 30%, BLS 24% adjusted no difference	no difference between groups
31	Kurola et al. 2002 Finland	expert panel all prehospital patients, specialist appraisal, ALS-PHYSICIAN n = 206	ALS-PHYSICIAN helicopter and GA	mortality 10,6%, no compatison, specialist appraisal	1,5% of patients benefit of ALS-treatment, 20.4% partial benefit
32	Bjerre et al. 2002 Danmark	chronic pulmonary disease ALS n = 67, BLS n = 72	ALS-PHYSICIAN, BLS-EMT ALS GA, BLS GA	mortality: ALS 15%, BLS 24%	ALS-PHYSICIAN better survival than BLS-EMT
33	Thomas et al. 2002 Massachusetts, U.S.A.	retrospective blunt trauma, ALS-PARAMEDIC helicopter n = 2292 ALS-PARAMEDIC GA, n = 3245, BLS-EMT GA n = 7723	3 groups: ALS-PARAMEDIC GA, ALS-PHYSICIAN helicopter, BLS-EMT GA	mortality: 9,4% (ALS helicopter or GA), BLS 3,0%; helicopter vs GA: OR 0,756 (0,59-0,98), BLS vs ALS 0,42 (0,32-0,56)	higher mortality in ALS than BLS higher mortality in GA than helicopter
34	Lossius et al. Norway 2002	expert panel all prehospital patients, ALS n = 1062 appraisal by specialist group, BLS no comparison material	ALS-PHYSICIAN 40% helicopter transport, 60% GA	mortality 20,7%, specialist appraisal 7% (n = 74) benefit fromALS-care	ALS useful, no controls
35	Lee et al. 2002 Australia	retrospective blunt trauma, head injury ALS-PARAMEDIC n = 1167 ALS-PHYSICIAN n = 224 BLS level 3 n = 452 BLS level 4 n = 45 BLS other n = 96	ALS-PHYSICIAN ALS-PARAMEDIC BLS-EMT (2 different levels) ALS GA, BLS GA	mortality: ALS-PARAMEDIC 24,8%, ALS-PHYSICIAN 19,6% BLS level 3 12,2%, BLS level 4 13,3%, BLS other 21% Adjusted: BLS OR = 1 ALS-PHYSICIAN OR = 4,27 ALS-PARAMEDIC OR = 2,18	higher mortality in ALS higer mortality in ALS-PHYSICIAN than in ALS-PARAMEDIC
36	Cristenzen et al. 2003 Danmark	retrospective before-after -design all prehospital patients ALS-PHYSICIAN n = 795+35 BLS-EMT n = 4989. before-after -study: in the second phase 28% of cases treated by ALS	ALS-PHYSICIAN BLS-EMT ALS GA BLS GA	phase I mortality 10,0% mortality in phase II = 10,5% phase II mortality in ALS-group 14,7%, phase II mortality in BLS-group 8,9% (p < 0,001) OR 1,06 (NS)	total mortality same in both periods
37	Osterwalder 2003 Switzerland	prospective TRISS multiple trauma ALS n = 196 BLS n = 71	ALS-PHYSICIAN, BLS-P, BLS-EMT EMT lower level education ALS GA or helicopter, BLS GA	mortality in ALS 11,2% BLS 14,1% (NS) predicted mortality in ALS 23,3%, actual mortality 22% predicted mortality in BLS 6,6% actual mortality 10%	ALS trend to lower mortality than BLS
38	Bochiccio et al. 2003 Maryland, U.S.A.	prospective retrospective brain injury: blunt (92%), penetrating (8%), comparison between patients intubated on site and those intubated in hospital intubated on site n = 78 intubated in hospital n = 113	all ALS-PARAMEDIC 67% had helicopter transport, others with GA	mortality: intubated on site 23% intubated in hospital 12,4% (p = 0,005)	higher mortality in patients intubated on site
39	Liberman et al. Canada, 2003	prospective epidemiological study all traumas Montreal ALS n = 801 Montreal BLS n = 4295 Toronto ALS n = 1000 Toronto BLS n = 1530 Quebec BLS n = 1779	Montreal ALS-PHYSICIAN Montreal BLS-EMT Toronto ALS-PARAMEDIC Toronto EMT-BLS Quebec BLS-EMT ALS GA, BLS GA	ALS 29% ISS 25-49 30% ISS 50-76 79% BLS 18% ISS 25-49 26% ISS 50-76 76% ALS-PHYSICIAN vs BLS 1,36* ALS-PARAMEDIC vs BLS 1,06**, ALS-PHYSICIAN vs ALS-PARAMEDIC 1,20** ALS vs BLS 1,21*, *p = 0,01 **p = NS	higher mortality in ALS
40	Danchin et al. 2004 France	retrospective chart review PHT n = 180 hospital trombolysis n = 365 CABG, PCI n = 434 no reperfusion n = 943	96% of PHT-patients got treatment from"mobile intensive care unit" all transported by GA	PHT 6% (1 year mortality) hospital thrombolysis 11% PCI 11%, no reperfusion treatment 21%, PHT mortality RR 0,49 (0,24 - 1,00)	lowest mortality in PHT Comparison between PHT and other reperfusion treatment RR = 0,52 (p = 0,08)
41	Biewener et al. 2004 Germany	prospective TRISS multiple trauma n = 403, 4 groups 1) HEMS-UNI n = 140 2) AMB-REG n = 102 3) AMB-UNI n = 70 4) INTER n = 91	all four goups ALS-PHYSICIAN 1) university hospital 2)regional hospital 3) university hospital 4) local hospital 1)transport by helicopter 2-4) transport by a GA	mortality rates: 1) 22,1% 2) 41,2% 3) 15,7% 4) 17,6% adusted risk 1) OR = 1 2) OR = 1,06 NS 3) OR = 4,06, p < 0,05 4) OR = 1,28, NS	ALS-PHYSICIAN + helicopter transport to university hospital is better than transport by a GA to regional hospital no difference in mortality between HEMS-UNI and AMB-UNI
42	Stiell et al. 2004 Canada	before-after -design cardiac arrest ALS n = 4247 BLS n = 1391	ALS-PARAMEDIC BLS-EMT ALS GA, BLS GA	mortality ALS 95,0% BLS 94,9% (p = 0,83) no adjustment No difference in QoL or cerebral performance	No difference in mortality.
43	Frankema et al. 2004 Netherlands	retrospective all serious injuries ALS n = 107 BLS n = 239	ALS-PHYSICIAN BLS-EMT, ALS helicopter, BLS GA	mortality: ALS 34,5%, BLS 24,3% adjustment: patients treated by ALS 2,4 fold probability to survive (p = 0,076). Blunt trauma OR 2,8, p = 0,036, penetrating trauma 0,2 (NS)	ALS better survival
44	Wang et al. 2004 Pennsylvania, U.S.A.	retrospective epidemiological study brain injury, comparison between patients intubated prehospitally with patients intubated in the hospital intubation on-site n = 1797 intubated in a hospital n = 2301	on-site intubation by ALS-PARAMEDIC or by ALS-PHYSICIAN, transportation by helicopter or by a GA	mortality on-site intubaltion 48,5%, hospitla intubation 28,2%, adjusted OR 3,99 (3,21-4,93)	patients intubated on-site had 4-fold risk of dying; patients intubated by using medication showed better survival.
45	Cameron et al. 2005 Australia	before-after-design all prehospital patients ALS-PHYSICIAN n = 211 ALS-PARAMEDIC BLS n = 163	ALS-PHYSICIAN, ALS-PARAMEDIC no BLS-group. ALS-PHYSICIAN helicopter ALS-PARAMEDIC helicopter	30 days mortality ALS-PHYSICIAN 2,8% ALS-PARAMEDIC 2,5%, NS	no difference bewtween ALS-PHYSICIAN and ALS-PARAMEDIC -groups
46	Mellado Vergel et al. 2005 Spain	retrospective cardiac infarct, PHT PHT n = 152 (ALS), hospital trombolysis (BLS) n = 829	ALS-PARAMEDIC BLS-EMT ALS GA, BLS GA	30 days mortality ALS 5,9%, BLS 26,6% (p = 0,066)	ALS (PHT) showed a trend to lower mortality
47	Di Bartolomeo et al. 2005 Italy	prospective traumatic cardiac arrest (blunt trauma) ALS n = 56, BLS n = 73	ALS-PHYSICIAN BLS-EMT+BLS-nurse ALS helicopter, BLS GA	ALS 96,5% only two patients survived BLS 100%, NS	no difference between ALS and BLS groups. prognosis still very poor
48	Davis et al. 2005 California, U.S.A.	retrospective epidemiological study brain injury ALS-helicopter n = 3017 ALS- GA n = 7295	Helicopter manned by paramedic, physician or nurse, ambulances manned by paramedics ALS helicopter, ALS GA	mortality: ALS helicopter 25,2% ALS ground ambulance 25,3% Adjusted OR 1,90 (1,60-2,25) mortality of patients intubated on site: ALS-helicopter 42,5% ALS-GA 43,1%, OR 1,42 (1,13-1,78)	ALS + helicopter + intubation on site better than ALS +GA + intubation in hospital
49	Björklund et al. Sweden, 2006	prospective prehospital trombolysis ALS n = 1690 BLS n = 3685 comparison between PHT entered in ambulance and thrombolysis in hospital	ALS-PARAMEDIC BLS-EMT, GA in both groups	mortality: ALS 5,4%, BLS 8,3 p < 0,001. ALS 0,71 (0,55-0,92) (1 year mortality); ALS 0,79 (0,61-1,03) 30 day mortality	ALS showed lower mortality
50	Sukumaran et al. 2006 Scotland	prospective TRISS all trauma patients ALS n = 12339 BLS n = 9078	ALS-PARAMEDIC BLS-EMT ALS GA, BLS GA	mortality: ALS 5,3%, BLS 4,5% p = 0,07; after adjustment no difference between groups	no difference between ALS and BLS groups
51	Iirola et al. 2006 Finland	retrospective before-after multiple trauma ALS n = 81, BLS n = 77	ALS-PHYSICIAN, BLS-EMT ALS helicopter (60%) or GA (39%) BLS GA	mortality: ALS 31%, BLS 18% p = 0,065; TRISS: material does not fit with MTOS-material QoL: no difference between groups	no difference between ALS and BLS groups, trend to bigger mortality in ALS-group (p = 0,065)
52	Klemen and Grmec 2006 Slovenia	prospective, historical controls multiple trauma, isolated head injury ALS n = 64, BLS n = 60	ALS-PHYSICIAN, ALS-EMT ALS GA, BLS GA	mortality ALS 40%, BLS 42% (NS). GOS level 4-5 achieved: ALS 53%, BLS 33%, p < 0,01	no difference in mortality in ALS better QoL
53	Stiell et al. 2007 Canada	prospective before-after dyspnoea, ALS n = 4218, BLS n = 3920	BLS-EMT, ALS-PARAMEDIC ALS GA, BLS GA	ALS 11,3% BLS 13,1% (p = 0,01)	lower mortality in ALS
54	Woodall et al. 2007 Australia	retrospective cardiac arrest ALS n = 1687 BLS n = 1288	ALS-PARAMEDIC BLS-EMT ALS GA, BLS GA	mortality: ALS 93,3%, BLS 95,3%; probablility for survival in all patients BLS = 1, ALS = 1,43 (1,02-1,99)	lower mortality ALS
55	Ma et al. 2007 Taiwan	prospective cardiac arrest ALS n = 386 BLS n = 1037	ALS-PARAMEDIC, BLS-EMT ALS GA, BLS GA	mortality ALS 93%, BLS 95% (NS); survival in ALS adjusted OR 1,41 (0,85-2,32)	no difference between groups
56	Seamon et al. 2007 Pennsylvania, U.S.A.	retrospective patients going to immediate thoracotomy comparison between ALS or BLS (n = 88) and private transport by laymen n = 92	ALS-PARAMEDIC ori BLS-EMT, compared to transportation by laymen.	mortality ALS,BLS 92% private transport 82,6% in multivariate analysis prehospital procedures were an independent predictor of mortality	better survival in persons transported by laymen

**57**	Stiell et al. 2008 Canada	Before-after -design. 92% blunt trauma, (ISS > 12), age ≥ 16 years ALS n = 1494 BLS n = 1373 Only 72% of the patients were transferred directly to the trauma centers from the scene.	ALS-PARAMEDIC, GA. BLS-PARAMEDIC, GA. Endotracheal intubation (7%), iv fluid (12%) and drug administration during the latter period.	Mortality ALS 18,9%, BLS 18,2% (p = 0,65) In patients with GCS < 9 mortality ALS 49,1%, BLS 40,0% (p = 0,02)	Implemantation of ALS did not decrease mortality or morbidity. In more severely injured patients (GCS < 9), mortality was lower in the BLS group.

Abbreviations: ALS = advanced life support, BLS = basic life support, EMT = emergency medical technician, LOS = length of stay (in hospital), ISS = Injury severity scale/score, TRISS = Trauma Score - Injury Severity Score, HEMS = Helicopter emergency medical service, GA = ground ambulance. PHT = prehospital throbolysis, OR = odds ratio, RR = risk ratio, ALS-N = advanced life support - nurse, ISS = injury severity score. QoL = quality of life.

Of the 46 studies there was one randomized controlled trial [13], 15 prospective follow-up studies [19,20,24,30,32,37-39,42,45,47,50,53,55,57] while the rest had a retrospective design. In the randomised trial [13] the effectiveness of prehospital thrombolysis and in-hospital thrombolysis for acute myocardial infarction was compared. Only two of the prospective studies reported an acceptable follow-up of patients [38,47].

### Studies concerning all patient groups

Five of the included studies [23,31,34,36,45] made no distinction between surgical, internal or other patients. None of those articles were considered to be of high quality. The reported result was the same in three articles [23,36,45]: No difference between ALS and BLS was found. Two articles [31,34] used a specialist group to assess the effectiveness of ALS without comparison to BLS.

### Prehospital thrombolysis for myocardial infarction

Two studies concerning thrombolytic treatment of cardiac infarction were published in 1995 [12,13] with three further articles on the topic published some ten years later [40,46,49]. In the two studies of 1995, patients having thrombolytic treatment showed a trend to better survival than patients having thrombolysis given in a hospital, but the result was not statistically significant.

In the three studies from 2004-2005, prehospital thrombolytic treatment was more effective than hospital thrombolysis, but only in Björklund's study [49] the result was statistically significant. The validity and generalisability of the studies were considered good.

### Cardiac arrest

The role of ALS in cardiac arrest was studied in nine studies [17,18,20,26,28,42,47,54,55]. In one study [54] ALS care showed better survival rates than BLS. In five studies patients treated by ALS and in one study patients treated by BLS showed a trend to better survival than patients in the control group, but the results were not statistically significant. One study was concentrated in traumatic cardiac arrest, with no difference between ALS and BLS [47]

### Penetrating and unselected traumas

We found eleven articles that dealt with penetrating or unselected trauma [16,19,21,22,25,27,39,43,50,56,57]. One included study [56] focused exclusively on penetrating trauma. Eight included studies considered both penetrating and blunt traumas, two of which [43,57] presented results separately for each type of injury.

No difference between ALS and BLS was found in five of these studies [19,21,25,50,57], though injuries in those studies were relatively mild (ISS over 15 in 11-15% of patients). Five of the studies showed better results for BLS [16,22,27,39,56]. In studies by Seamon [56] and Demetriades et al. [16], ALS treatment of trauma was compared with transportation to hospital by laymen. In both studies, transportation by a layperson showed better survival rates than ALS.

Three studies [22,27,39] showed better survival in BLS patients as well as the study by Stiell et al [57] among subgroup of patients with GCS < 9. In the study by Frankema et al. [43] blunt trauma patients having ALS treatment given by a physician and transported by a helicopter showed better survival than BLS-treated patients transported by a GA.

In general, there is no evidence that ALS would be superior compared to BLS in penetrating or unselected traumas. There is one study supporting ALS by Frankema et al. [43] but the result was confounded by the transportation method.

### Blunt head injury

Six studies concentrated in blunt head injury [24,29,30,38,44,48]. In three studies the combination of ALS treatment and helicopter transportation gave better results than BLS with a GA [24,29,48]. In two studies [38,44] intubation without medication by a paramedic was harmful compared to intubation in a hospital by a physician using medication to assist intubation. In the study by Di Bartolomeo [30], there was no difference between ALS and BLS.

### Multiple blunt injury

Eight studies concerned patients with multiple blunt injuries [33,35,37,41,47,51,52,57]. No clear difference between ALS and BLS was found [47,51,52,57]. Two studies showed better results for BLS [33,35]. Results were difficult to estimate, because the comparison between ALS and BLS was confounded by transportation (helicopter or GA) and treatment level of the hospital [41].

### Respiratory distress

The effect of prehospital treatment for patients having respiratory distress was studied in two papers [32,53], both of them favouring ALS.

### Other diseases

One study was focused on epileptic patients [14] and one study on all unconscious patients (epilepsy, hypoglycaemia or stroke) [15]. In epileptic emergencies, the results favour ALS [14], and in the other study no difference was detected [15]. Hardly any research exists on several patient groups needing emergency care (e.g. stroke, intoxication, drowning,).

## Discussion

The most remarkable limitation in this study is that definition of ALS and BLS is changing in time and place. This main problem is followed by several other problems:

1. Both ALS and BLS have developed and some methods used formerly in ALS may later be included into BLS.

2. Different studies use also different definitions of ALS and BLS.

3. The inclusion and exclusion criteria of this study have been set according to one definition. If the definition is changed, the set of articles may also be different.

4. The level of credibility of separate articles is always subjective, especially in articles with border-line credibility.

In studies concerning unselected patient groups, the evidence does not support ALS. Similarly, in studies of all injuries, ALS has not been found to be superior compared to BLS. ALS treatment by a paramedic can even be harmful compared to BLS. When the patient material has concentrated to more serious cases, blunt head injuries or multiple injuries, some studies have demonstrated a beneficial effect of ALS. However, not all studies confirm this. ALS seems to be most beneficial when having an experienced physician in the staff. The critical limit of the experience of the staff can be defined as the ability to perform an intubation by using hypnotics and muscle relaxants. If this limit has not been reached, an ALS activity can be harmful.

We did not find evidence supporting ALS in regard to cardiac arrest if use of a defibrillator is included into BLS. In cardiac arrest, early cardiopulmonary resuscitation (CPR) and defibrillation are essential for a patient's survival. For instance in Finland, even lay rescuers are trained to use an automated external defibrillator and start CPR. No evidence has been found on the effectiveness of other activities in treating sudden cardiac arrest when the end point of the studies has been secondary survival (at discharge from hospital). The most promising intervention - prehospitally initiated therapeutic hypothermia - still needs more validation.

Prehospital thrombolysis seems to be superior to thrombolysis given in a hospital, but it should be remembered that prehospital thrombolysis is only one link in the treatment chain. We have not assessed the effect of other treatments for acute myocardial infarction.

We interpret the contradictory findings between studies as due mainly to the multitude of definitions for ALS and BLS, differences in treatment populations and interventions, the high risk of bias in the original studies, and lack of statistical power: cases where ALS is beneficial may be too rare to be identified by statistical methods in an unselected material. There is a clear need for international definitions of ALS and BLS, appropriate documentation of populations and performed interventions in trials, and more high quality studies taking into account different patient groups. Among defined patients, e.g. those having brain injuries, the benefit of ALS can be found if there are enough eligible patients. The infrequency of potential cases for ALS indicates that an ALS emergency system requires a large enough catchment area.

Only a very few studies analyzing prehospital care of trauma described the severity of trauma and the interventions performed. The control groups were usually not comparable to the index group: the ALS groups tend to consist of more severely injured patients. Additionally, common confounding factors include different transportation means (helicopter vs. ground ambulances) and the different levels of the admitting trauma hospitals. In many studies patients with only very mild injuries or, conversely, patients with little chance of survival (i.e. traumatic cardiac arrest or gunshot wounds to head) have been included.

The majority of studies favour the use of a helicopter, but the results were contradictory. In many studies, the effectiveness of an operation model (ALS vs. BLS) was confounded by not being able to take into account the vehicle used (helicopter vs. ambulance).

The effectiveness of a helicopter may warrant that it is used for all patient groups, and especially for patients where prehospital treatment is known to be effective (e.g. myocardial infarction). Among trauma patients, the best results are probably achieved when the severity of the trauma can be classified as being moderate or serious but not indicating a poor prognosis. A helicopter service requires a sufficiently large catchment population.

The distance from the site to the hospital mediates the effectiveness of ALS. For short distances (urban and semi-urban areas), there is no evidence favouring ALS in the case of an injury. According to Nicholl et all [58] a 10-km increase in distance from a hospital is associated with a 1% absolute increase in mortality. For longer distance, ALS operating with a helicopter seems to be effective, but still there is a contradiction in terms of cost-effectiveness: longer distances are associated with a more sparse population. In a British study concerning trauma patients only [7], the population base was estimated to be 3 million. In Finland, injuries make only about 20% of all cases in a typical Finnish Helicopter Emergency Medical Service. Thus the estimation by Nicholl is in line with a Finnish practical experience that a population of 0.5 million to be reached in 30 minutes may be satisfactory for a helicopter-ALS -service.

While the concepts "scoop and run" or "stay and play" are often present in the literature, they reflect the tactics of emergency services, and should not be seen to correlate directly with ALS or BLS. An ALS-level emergency unit employs rapid assessment and scoop and run -tactics when a definitive treatment outside the hospital is not possible. A penetrating injury with bleeding is an example of a situation where immediate surgical treatment is imperative.

When a diagnosis is feasible and definitive, an effective treatment can be started prehospital by using stay and play -tactics, for example, prehospital thrombolysis in a myocardial infarct and early defibrillation in a cardiac arrest.

The tactic of choice is determined by the nature of the emergency, the available services, and the possibilities for starting the treatment in the hospital. All hospitals can not give all treatments at all times of the day. Treatment delays are caused not only by distances but also by the care level of the hospital.

The right to emergency health care services in many countries (including Finland) is granted to citizens by a legal constitution. Equal access to effective treatment is also one of the fundamental principles of health care in many countries. How to organize an emergency service is affected by medical knowledge but also by general opinion, people's sense of safety and earlier structures of services. It may be reasonable to organize ALS-level emergency services even when there is uncertainty about the cost-effectiveness of such services. An ALS system operating with a helicopter can also bring services to sparsely populated areas.

## Conclusions

The overall quality of the analyzed studies was poor. Among 1333 studies, only one randomised controlled trial was found. Thus, no conclusions on the effectiveness of advanced prehospital care in unselected patient cohorts can be drawn.

In the prehospital care of sudden cardiac arrest early defibrillation and cardiopulmonary resuscitation are still essential; further ALS interventions have not been able to demonstrate increased survival. Prehospitally initiated hypothermia is a promising treatment but is not as yet an evidence-based intervention.

Prehospitally initiated thrombolysis of myocardial infarction improves survival when compared with in-hospital initiation of thrombolytic treatment.

There is evidence that ALS is beneficial in epileptic patients and among those with respiratory distress.

Due to multiple methodological problems found in trauma studies, the comparison of ALS and BLS prehospital care is difficult and, in unselected trauma cohorts, impossible. It seems obvious that in urban settings and in patients with penetrating injuries, ALS does not improve survival. In some patients, for instance, patients with severe head injuries, ALS provided by paramedics and intubation without anaesthesia can even be harmful due to uncontrolled intracranial pressure. If the prehospital care is provided by an experienced physician and by a HEMS organisation (Helicopter Emergency Medical Service), ALS interventions may be beneficial for patients with multiple blunt injuries.

In many patient groups such as patients with cerebrovascular problems, intoxication, drowning etc. there is very little research available on the effectiveness of ALS and BLS levels of prehospital care and thus no conclusions can be made.

The need for high quality controlled clinical studies is obvious. Besides that, the development of prehospital care requires uniform and full documentation and follow-up of patients as well as register studies based on real-life data.

## Competing interests

JR is a doctor-in-charge of MediHeli helicopter emergency medical service, Helsinki, Finland. TI is a clinician of MediHeli 02, Turku, Finland. Other authors declare that they have no competing interests.

## Authors' contributions

OPR, TI, JR, HP and AM all have been participating in the study design and planning the search strategy. OPR, TI, JR and AM assessed the articles and made the data extraction. The manuscript was completed by OPR, TI and JR, assisted by AM. All authors have read and approved the final manuscript.
